# Geotemporospatial and causal inferential epidemiological overview and survey of USA cannabis, cannabidiol and cannabinoid genotoxicity expressed in cancer incidence 2003–2017: part 3 – spatiotemporal, multivariable and causal inferential pathfinding and exploratory analyses of prostate and ovarian cancers

**DOI:** 10.1186/s13690-022-00813-6

**Published:** 2022-03-30

**Authors:** Albert Stuart Reece, Gary Kenneth Hulse

**Affiliations:** 1 Division of Psychiatry, University of Western Australia, Crawley, WA 6009 Australia; 2 School of Medical and Health Sciences, Edith Cowan University, Joondalup, WA 6027 Australia; 3 Brisbane, Australia

**Keywords:** Cannabis, Cannabinoid, Δ9-tetrahydrocannabinol, Cannabigerol, Cannabidiol, Mechanisms, Congenital anomalies, Oncogenesis, Genotoxicity, Epigenotoxicity, Chromosomal toxicity, Multigenerational genotoxicity, Transgenerational teratogenicity, Dose-response relationship, Supra-linear dose response, Sigmoidal dose-response

## Abstract

**Background:**

The epidemiology of cannabinoid-related cancerogenesis has not been studied with cutting edge epidemiological techniques. Building on earlier bivariate papers in this series we aimed to conduct pathfinding studies to address this gap in two tumours of the reproductive tract, prostate and ovarian cancer.

**Methods:**

Age-standardized cancer incidence data for 28 tumour types (including “All (non-skin) Cancer”) was sourced from Centres for Disease Control and National Cancer Institute using SEER*Stat software across US states 2001–2017. Drug exposure was sourced from the nationally representative household survey National Survey of Drug Use and Health conducted annually by the Substance Abuse and Mental Health Services Administration 2003–2017 with response rate 74.1%. Federal seizure data provided cannabinoid concentration data. US Census Bureau provided income and ethnicity data. Inverse probability weighted mixed effects, robust and panel regression together with geospatiotemporal regression analyses were conducted in R. E-Values were also calculated.

**Results:**

19,877 age-standardized cancer rates were returned. Based on these rates and state populations this equated to 51,623,922 cancer cases over an aggregated population 2003–2017 of 124,896,418,350. Inverse probability weighted regressions for prostate and ovarian cancers confirmed causal associations robust to adjustment. Cannabidiol alone was significantly associated with prostate cancer (β-estimate = 1.61, (95%C.I. 0.99, 2.23), *P* = 3.75 × 10^− 7^). In a fully adjusted geospatiotemporal model at one spatial and two temporal years lags cannabidiol was significantly independently associated with prostate cancer (β-estimate = 2.08, (1.19, 2.98), *P* = 5.20 × 10^− 6^). Cannabidiol alone was positively associated with ovarian cancer incidence in a geospatiotemporal model (β-estimate = 0.36, (0.30, 0.42), *P* <  2.20 × 10^− 16^). The cigarette: THC: cannabidiol interaction was significant in a fully adjusted geospatiotemporal model at six years of temporal lag (β-estimate = 1.93, (1.07, 2.78), *P* = 9.96 × 10^− 6^). Minimal modelled polynomial E-Values for prostate and ovarian cancer ranged up to 5.59 × 10^59^ and 1.92 × 10^125^. Geotemporospatial modelling of these tumours showed that the cannabidiol-carcinogenesis relationship was supra-linear and highly sigmoidal (*P* = 1.25 × 10^− 45^ and 12.82 × 10^− 52^ for linear v. polynomial models).

**Conclusion:**

Cannabinoids including THC and cannabidiol are therefore important community carcinogens additive to the effects of tobacco and greatly exceeding those of alcohol. Reproductive tract carcinogenesis necessarily implies genotoxicity and epigenotoxicity of the germ line with transgenerational potential. Pseudoexponential and causal dose-response power functions are demonstrated.

**Supplementary Information:**

The online version contains supplementary material available at 10.1186/s13690-022-00813-6.

## Background

Cannabis has been linked with cancers at many sites including head and neck, brain, lung, larynx, prostate, testis, cervix and urothelium by previous studies [1–18]. However uncertainty on many of these points persists as other studies with conflicting results also appear both in the literature [4, 19, 20] and in reviews [16, 21–24].

The most strongly documented link between cannabis and cancer is for testicular cancer where several recent studies have confirmed an association [3, 8–10] and dose-response effects have been demonstrated [3, 8, 10]. Endocrine disruption through such events as low birthweight, short gestation, tall stature, maternal bleeding, twinship, first position in the sibship and small sibship has also been linked with the development of testicular cancer. Since the testis houses the male germ cell epithelium it is conceivable that genomic or epigenomic damage incurred by the male germ cells may be passed along to subsequent generations.

This possibility is confirmed by published reports linking prenatal cannabis exposure with paediatric cancer incidence including rhabdomyosarcoma [16], childhood neuroblastoma [15] and leukaemia particularly non-lymphoblastic leukaemia [17, 19] which together demonstrate evidence of inheritable mutagenicity and carcinogenicity in human populations [25, 26]. The importance of mutagenicity, carcinogenicity and heritability was underscored by a recent report showing that breast, thyroid, liver and pancreatic cancers and acute myeloid leukaemia along with three chromosomal trisomies (21, 18 and 13), Turners syndrome and Deletion 22q11.2 were increased causally and across space-time in relation to cannabis use [27]. Other reports show that cannabis exposure is a likely cause and driver of rising paediatric cancer rates [28] including the commonest childhood cancer acute lymphoid leukaemia [28].

Cannabidiol is of particular concern as it is often thought to be relatively safe, is widely available in many jurisdictions and its known genotoxicity [29–37] and epigenotoxicity [38–48] is generally unknown and ignored.

Prostate cancer was previously found to be greatly elevated by current cannabis exposure with an odds ratio of 4.7 (95%C.I. 1.4, 15.5) [7]. Intriguingly endocrine disruption was identified as one possible mechanism to explain this relationship [7]. Cannabis is a well established endocrine disruptor [49–59]. Whilst there are no extant papers documenting the relationship of cannabinoid exposure to ovarian cancers oocytes have been shown to be highly sensitive to cell death during cell division under the influence of cannabinoids [60] and the ovary is also known to be highly sensitive to inhibitors of mitochondrial metabolism a role which several cannabinoids including cannabidiol have long been known to play [36, 37, 61–65].

Earlier reports in this series have considered the impact of substance and cannabinoid exposure on a panel of 28 common cancers across USA [66, 67]. Prostate and ovarian cancer were found to be particularly associated with cannabidiol exposure in these bivariate studies [66, 67]. It was the purpose of this paper to investigate this relationship further in a multivariable context using the tools of causal inferential and geospatial modelling and to examine the impacts of limited mathematical modelling on some of the important models to proceed from these regression studies. This is done both to provide detailed information on these two tumours and to demonstrate an analytical and causal inferential pipeline for the further exploration of such rich epidemiological datasets.

## Methods

### Data

Rates of age-adjusted cancer rates by state and year and cancer type was taken from the Surveillance, Epidemiology and End Results (SEER) database from the Centres for Disease Control (CDC) Atlanta, Georgia and the National Cancer Institute (NCI) and from the National Program of Cancer Registries (NPCR) and SEER Incidence US Cancer Statistics Public Use Database 2019 submission covering years 2001–2017 using the SEER*Stat software [68]. The focus of this study was 28 of the most common cancers (as listed below). This includes the category all non-skin cancer (called All Cancer in this report). This was joined with drug use cross-tabulation data across USA by state and year from the National Survey of Drug Use and Health (NSDUH) Restricted-Use Data Analysis System (RDAS) of the Substance Use and Mental Health Data Archive (SAMHDA) held by the Substance Use and Mental Health Services Administration (SAMHSA) 2003–2017 [69]. Thus the overlap period between the cancer and drug exposure datasets was 2003–2017 which therefore became the period of analysis. The variables of interest were last month cigarettes, last year alcohol use disorder (AUD), last month cannabis, last year non-medical use of opioid analgesics (Analgesics) and last year cocaine. Quintiles of substance exposure were calculated for each year numbered from one, the lowest quintile, to five the highest exposure quintile. Data on median household income, ethnicity and population by state and year was sourced directly from the US Census bureau via the tidycensus package [70] in R including linear interpolation for missing years. The ethnicities of interest were Caucasian-American, African-American, Hispanic-American, Asian-American, American Indian / Alaska Native (AIAN) and Native Hawaiian / Pacific Islander (NHPI). Data on cannabinoid concentration across USA was taken from reports published by the US Drug Enforcement Agency (DEA) for the five cannabinoids Δ9-tetrahydrocannabinol (THC), cannabigerol (CBG), cannabichromene (CBC), cannabinol (CBN), and cannabidiol (CBD) [71–73]. It was multiplied by state level cannabis use to provide an estimate of state level exposure. Quintiles of cannabinoid exposure were calculated on the whole period considered in aggregate. Age adjusted case numbers were derived by multiplying the age-adjusted cancer rate in each state and year by the population of that state and dividing it by 10,000.

### Statistical analysis

Data was processed in R-Studio version 1.3.1093 (2009–2020) based upon R version 4.0.3 (2020-10-10). Covariates were log transformed guided by the Shapiro-Wilks test. Data was manipulated using the “dplyr” package in the “tidyverse” [74]. Graphs were drawn in ggplot2 from tidyverse [74, 75] and maps and graphs were drawn in R-Base, ggplot2 and “sf” (simple features) [76]. Some colour palettes employed the viridis and plasma palettes taken from the package “Viridis” [77] and several palettes were originally designed for this project. Bivariate maps were drawn using colorplaner two way colour matrices [78]. All maps and graphs are original and have not been previously published. General additive models (GAM) were computed using the package “mgcv” [79, 80]. Models were compared using the Anova test in R-base.

### Regression models

Bivariate linear trends were computed with linear regression from R-Base. Repeated measures mixed effects regression was conducted using the package “nlme” using state as the random effect [81]. Robust generalized linear regression was conducted in the R “survey” package again using state as the identity variable [82]. Panel regression was conducted using package “plm” using a space-time method [83]. In each case model reduction from initial to final models was by the classical method of serial deletion of the least significant term.

Geotemporospatial regression was conducted using the spreml (spatial panel random effects maximum likelihood) function from the “splm” (spatial panel linear modelling) package [84]. Spatial weights matrices describing the spatial relationship between states were computed from edge and corner (“queen”) relationships computed from the package “spdep” [85] and edited as described. Model specification was checked by the previously described reverse method [86]. Four spatial coefficients are calculated in full spatial panel random error maximum likelihood (spreml) models as phi, psi, rho and lambda corresponding to the terms for random effects, serial autocorrelation effects, spatial coefficient and autocorrelation of the spatial coefficients respectively [87]. When verifying model specification by the reverse method non-significant error terms are deleted from the fully specified (error = semsrre + lag) model [86]. This was the procedure used in the present report. Such procedures allow for fine control of the structure of the error terms.

Different forms of regression were used for the following reasons. Mixed effects modelling has the advantage over linear modelling that repeated measurements can be considered from the same region. Inverse probability weighting is possible in mixed effects, robust and panel modelling but not in spatial models. Mixed effects, panel and spatial models allow the calculation of a model standard deviation so E-Values can be calculated from such models. Lagging can be applied in panel and spatial panel models but not in mixed effects or robust models. Instrumental variables can be employed in panel models but not in spatial panel models. Spatial panel models allow the use of both spatially and temporally lagged variables as well as spatially and temporally lagged variables simultaneously. Hence it was felt that the use of several different regression model types would allow a broad and comprehensive overview of the analyses and allow result verification by several alternative methods.

### Simultaneous multiple model analysis

This was conducted in the tidyverse package “purrr” [74] using tidy and glance from package “broom” [88] using established nest-map-unnest workflows. In this way a whole long dataset providing data on many cancers could be analyzed in a single analysis run at one time.

### Causal inference

Causal inference was addressed in two ways. Firstly inverse probability weighting (IPW) was conducted on all mixed effects, robust and panel models which had the effect of equilibrating exposure across all observed groups. IPW were computed from the R-package “ipw” [89]. Inverse probability weighting transforms an observational dataset into a pseudo-randomized dataset so that it becomes appropriate to draw inferences as to truly causal relationships. Secondly E-values were computed using the R-package “EValue” [90] both from count data and from regression equations using the parameter estimate, its standard error and the standard model deviation [91–93]. E-Values were computed both for regression models and for the predicted output from fitted models. E-Values were computed for mixed effects, panel and spatial panel models [92–95]. Minimum E-Values above 1.25 are said to suggest causal relationships [91].

### Predictive spatial modelling

Selected spatial panel models were chosen for predictive analysis as described. Spatial panel (spreml) model objects include a vector of model predicted values ($fitted.values). Matrix multiplication was used to multiply 101 vectors, comprising percentiles zero to 100 of exposure to the cannabinoids THC, cannabigerol and cannabidiol by the model parameter coefficients to produce model predicted values. Terms which did not include cannabinoids were set at their mean value for this exercise and the intercept coefficient was set at one. In each case the resulting predictions were outside and below the range of the cancer incidence, which was unsurprising as the models themselves included both log and lag terms.

The z-transformation is often used in statistics to correct variable distributions. Subtracting the mean of a data series from the values and dividing by the standard deviation of that data series will change its mean to zero and its standard deviation to 1. This is a standard statistical transformation known as the z-transformation. In this case an extended z-transformation procedure was performed whereby the mean of the predicted data series for the cancer rate was added to the mean after z-transformation and the new standard deviation was set at the ratio of the median of the raw data series to the median of the fitted values from the model. Hence the final predicted value conversion formula appears as follows:

$$Recalibrated\_ Result=\left(\left( Res- mean(Res)\right)/\kern0.5em \left(\left( sd(Res)\right)/\left( sd(FVV)\ast \left( median\left( SPDSST\$ CancRt\right)\kern0.5em / median(FVV)\right)\right)\right)\right)+\kern0.5em \left( mean\left( SPDSST\$ CancRt\right)\right)$$

where Res is the raw results from matrix multiplication, mean is the average, sd is the standard deviation, median is the median, SPDSST is the spatial panel space-time dataset for the cancer concerned, FVV is the fitted values from the model, CancRt is the observed age-adjusted cancer rate for that tumour reported from SEER and $ is a placeholder for the dataframe signifying the variable name. The reported analysis of model predictions was performed on the Recalibrated Results after application of this extended z-transformation conversion formula.

### Spatially and temporally lagged modelling

As it is well known that there has been a spatiotemporal progression of the re-scheduling of cannabis products and availability across USA over the last decade it was of interest to see if accounting for spatially and temporally lagged effects affected the outcomes of the analyses or the main conclusions. Preliminary studies suggested that single spatial lags were appropriate. Cancer is also a time lagged disease so there were several reasons for wanting to consider a series of temporal lags to investigate the effect that temporal lagging had on model progression. Temporal lagging was used in both panel and spatiotemporal models whilst spatial lagging was restricted to spatial models.

*P* < 0.05 was considered significant throughout.

### Data availability

Data, including R-code, ipw weights and spatial weights has been made freely available through the Mendeley Data repository online and can be accessed at 10.17632/dt4jbz7vk4.1

### Ethics

Ethical approval for this study was granted from the University of Western Australia Human Research Ethics Committee approval number on 7th January 2020 RA/4/20/7724.

## Results

The cancers upon which we chose to focus our attention were chosen because they were relatively common or because they involved tissues which had been implicated in the literature with cannabinoid activities. For this reason cancers of the male and female reproductive tract were well represented amongst the cancers chosen for this study. The list in alphabetical order comprises tumours of: acute lymphoid leukaemia (ALL), acute myeloid leukaemia (AML), bladder, brain, breast, cervix, chronic lymphoid leukaemia (CLL), chronic myeloid leukaemia (CML), colorectum, oesophagus, Hodgkins lymphoma, Kaposi sarcoma, kidney, liver, lung, melanoma, multiple myeloma, Non-Hodgkins lymphoma, oropharynx, ovary, pancreas, penis, prostate, stomach, testis, thyroid and vulva and vagina combined. Based on 2017 data the 27 cancers chosen comprehended 1,339,737 of the 1,670,227 cancers reported to state cancer registries in that year or 80.21% of all non-melanoma non-skin cancers reported. In addition total non-skin cancer was also included in this list making 28 cancer types in all.

19,877 age-adjusted cancer rates were retrieved from the SEER*Stat State NPCR database. The total age-adjusted number of cancers reviewed across the 28 cancer types was 51,623,922 and the total aggregated population across the period 2003–2017 was 124,896,418,350.

Other papers in this series consider these covariates as continuous [66] and categorical [67] covariates respectively.

### Specific cancer examples

**Fig. 1:**
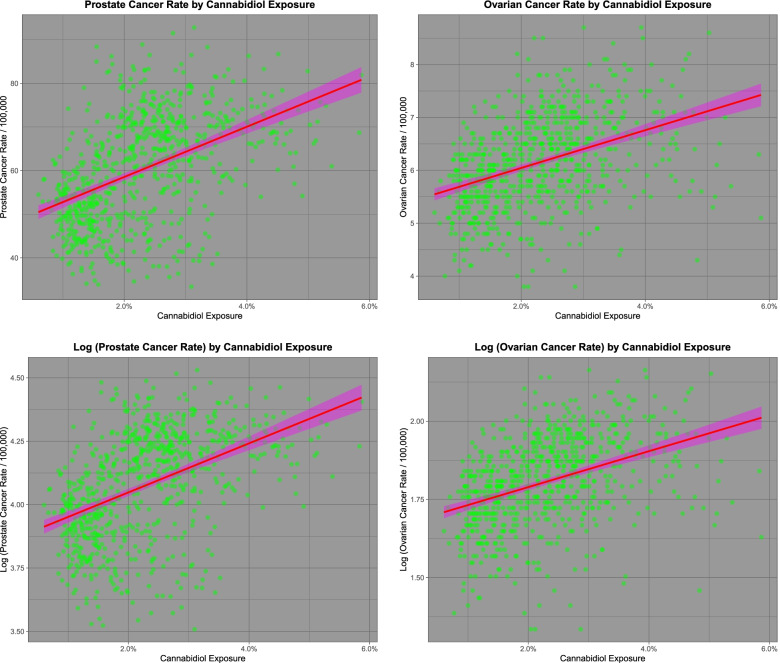
Relationship of prostate and ovarian cancer incidence to cannabidiol exposure

**Fig. 2:**
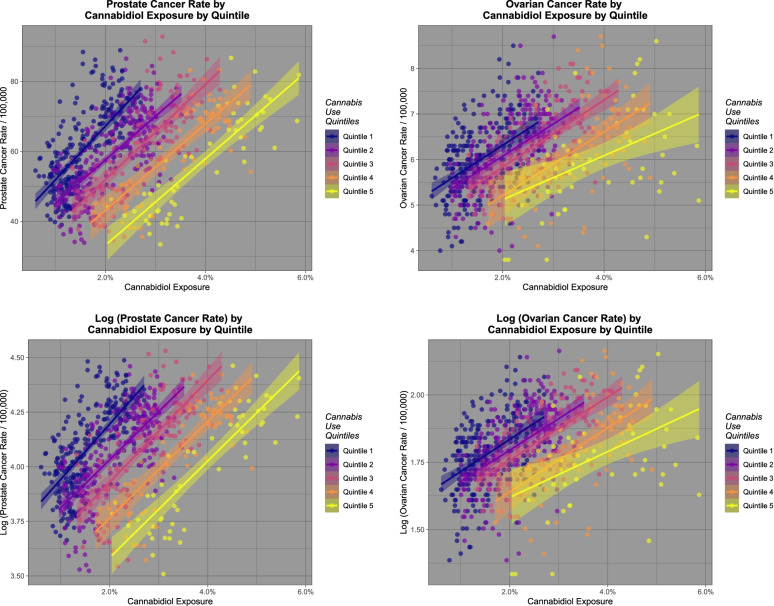
Relationship of prostate and ovarian cancer incidence to cannabidiol exposure by cannabidiol exposure quintile

Figure 1 shows the rates of two selected cancers, namely (A) prostate cancer and (B) ovarian cancer against cannabidiol use. Panels (C) and (D) show these same plots as log of the cancer rates. One notes that both prostate and ovarian cancer rates are falling, as is cannabidiol exposure (Figs. 1 and 2). Fig. 2 shows a similar plot to Fig. 1 but now representing the quintiles of cannabis exposure. The steady shift of the regression line to the right indicates an ordered relationship of these two tumours to cannabidiol exposure quintile. These tumours are analyzed in greater detail in the third paper in this series.

### Prostate cancer

It is of interest to investigate some of the tumours most significantly linked to cannabidiol exposure in further detail. For this purpose prostate and ovarian cancer have been chosen as illustrative rather than exhaustive examples of the way in which more detailed analyses may be conducted upon these datasets.

**Fig. 3:**
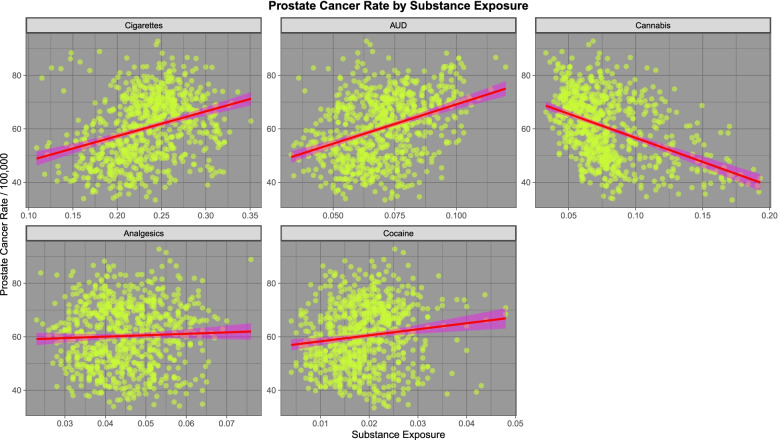
Prostate cancer rates by substance exposure

We turn first to prostate cancer. The dramatically declining rate of prostate cancer was noted in the first Figure in the first paper in this series. This is likely related to the impact of the introduction of Prostate Specific Antigen (PSA) screening and its widespread application in the community with a falling impact thereafter. Figure 3 (in the present paper) sets out the relationship of prostate cancer to the exposure to various substances. One notes an obviously positive relationship with tobacco, alcohol and cocaine exposure and a negative relationship with cannabis exposure.

**Fig. 4:**
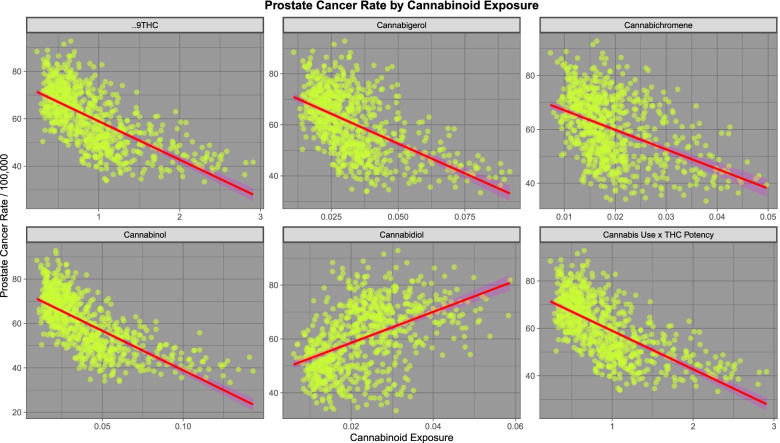
Prostate cancer rates by estimated cannabinoid exposure

Figure 4 shows the relationship of prostate cancer incidence to cannabinoid exposure. One notes that in most cases cannabinoids are negatively associated with prostate cancer incidence with the notable exception of cannabidiol which is positively associated.

**Fig. 5:**
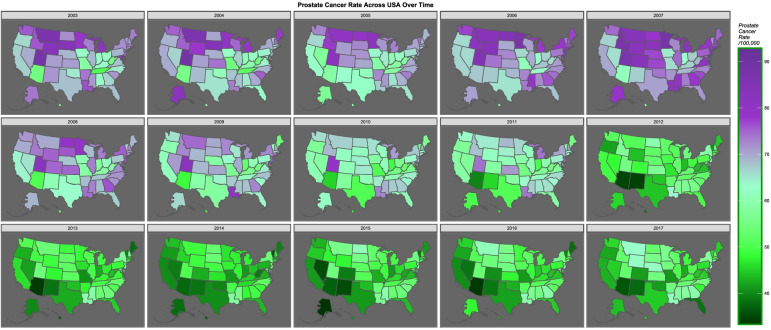
Map-graph of prostate cancer rates across the USA

**Fig. 6:**
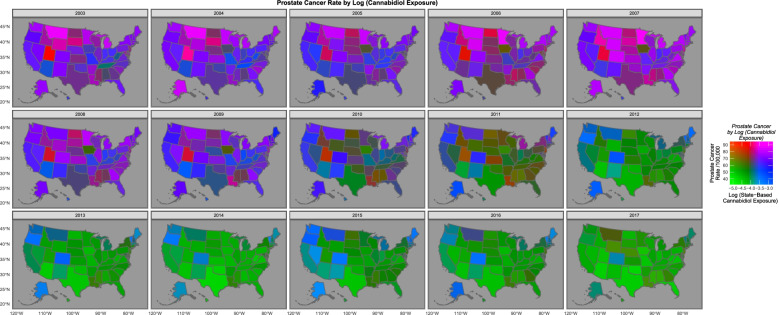
Map-graph of bivariate distribution of prostate cancer and cannabidiol exposure across the USA. Drawn using colorplaner palette

Figure 5 sets out map-graphically the declining rate of prostate cancer across USA over time. Figure 6 is a bivariate map plot of the relationship between prostate cancer incidence and cannabidiol exposure. The purple and pink tones show where both cannabidiol and prostate cancer are high. One notes that as both fall the map changes to green where both are low, with the sole exception of Maine, Vermont and New Hampshire which remain persistently elevated.

Supplementary Table 1 (Excel sheet “ST1 Pros lme”) shows a series of increasingly inverse probability weighted complex mixed effects models of the relationship of prostate cancer with various parameters. The relationship with cannabis, THC and cannabigerol is noted to be strongly negative. However the relationship with cannabidiol is highly significantly positive (β-estimate = 25.09, 95%C.I. 23.31, 26.87). The lower part of the Table presents final additive and interactive comprehensive models including all drugs, ethnicity and income

Supplementary Table 2 (Excel sheet “ST1 Pros lme Comp”) presents the results of an interactive cannabinoid model. In this model terms including cannabidiol are mostly negative

Supplementary Table 3 (Excel sheet “ST1 Pros SG”) presents the results of comprehensive additive and interactive inverse probability weighted robust generalized linear regression. In the additive model cannabidiol is independently significant and the coefficient is positive. The interactive model includes two terms where cannabidiol is positive and three where it is negative. The net effect of cannabidiol, and indeed of all cannabinoids in this interactive model, is strongly positive (by matrix multiplication)

Supplementary Table 4 (Excel sheet “ST1 Pros plm Intro”) shows the results of panel regression for increasingly complex models. Cannabis terms are negative in additive models. As shown in the last two models in this table in both additive and interactive models cannabidiol terms are positive

Supplementary Table 5 (Excel sheet “ST5 Pros plm Lag Add”) presents a series of additive panel models lagged to 0, 2, 4, 6, and 8 years. One notes that at zero, 4 and 6 years of lag cannabidiol is independently significant in these models and its terms are positive. However at eight years the term becomes negative. This indicates that the effects of cannabidiol appears to have dissipated at eight years which is to be expected of an environmental carcinogen

Supplementary Table 6 (Excel sheet “ST1 Pros plm IR”) presents the results of lagging interactive models at zero and two years. Due to the technical requirements of panel models and the restrictions imposed by interactions on dimensionality constraints exhaustive analysis in this format is not possible

### Spatiotemporal models of prostate cancer

**Fig. 7:**
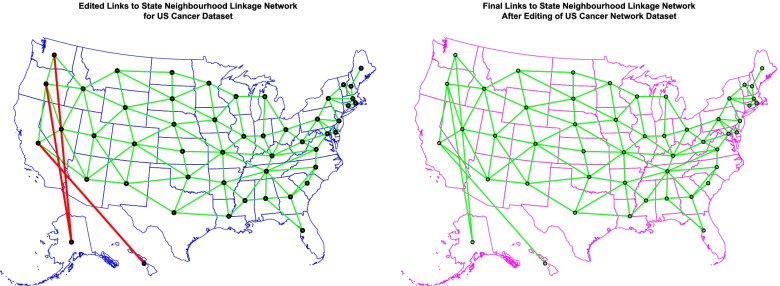
Geospatial links between various US states (**A**) edited and (**B**) Final. These links were used to form the sparse spatial weights matrices used in the geospatial models for prostate and ovarian cancer

Figure 7 presents the geospatial relationships between the various US states. As shown Hawaii and Alaska were conceptually elided and edited onto the contiguous continental 48 US states to facilitate geospatial modelling.

Table 1 shows the introductory results of geospatial modelling with these data. Cannabidiol is again found to be strongly associated with prostatic cancer rates across space and time together (β-estimate = 1.61 (C.I. 0.99, 2.23), *P* = 3.75 × 10^− 7^).

**Table 1:** Prostatic Cancer – Introductory Space – Time Models

Parameter			Model		
Parameter	Estimate (C.I.)	P	Coefficient	Value	***P***-Value
***Cannabis Alone***			S.D.	4.8855	
***spreml(Cancer Rate ~ Cannabis)***			Log.Lik	− 2104.454	
Cannabis	−3.34 (−4.54, − 2.15)	4.36e-08	phi	1.799117	0.0003
			psi	0.662222	< 2.2e-16
			rho	−0.809768	< 2.2e-16
			lambda	0.902303	< 2.2e-16
***THC Alone***			S.D.	5.0538	
***spreml(Cancer Rate ~ THC exposure)***			Log.Lik	− 2099.24	
THC exposure	−2.06 (− 2.59, − 1.52)	4.48e-14	phi	1.968582	0.0021
			psi	0.626775	< 2.2e-16
			rho	−0.782918	< 2.2e-16
			lambda	0.887035	< 2.2e-16
***Cannabigerol Alone***			S.D.	1.0251	4.9450
***spreml(Cancer Rate ~ Cannabigerol exposure)***			Log.Lik	− 979.9955	− 2106.9850
Cannabigerol exposure	− 1.84 (− 2.58, − 1.1)	1.01e-06	phi	1.864451	0.0020
			psi	0.655393	< 2.2e-16
			rho	−0.80251	< 2.2e-16
			lambda	0.906276	< 2.2e-16
***Cannabidiol Alone***			S.D.	5.1501	
***spreml(Cancer Rate ~ Cannabidiol exposure)***			Log.Lik	− 2105.8960	
Cannabidiol exposure	1.61 (0.99, 2.23)	3.75e-07	phi	2.064829	0.0016
			psi	0.675325	< 2.2e-16
			rho	−0.782638	< 2.2e-16
			lambda	0.897849	< 2.2e-16
***Additive Model - Drugs***			S.D.	5.0551	
***spreml(Cancer Rate ~ Age + Cigarettes + AUD + Cannabis + Analgesics + Cocaine***			Log.Lik	− 2088.4810	
AUD	30.08 (3.65, 56.51)	0.02571	phi	2.045313	5.47e-05
Cannabis	−1.54 (−3, −0.08)	0.0384	psi	0.607328	< 2.2e-16
Age	−0.79 (− 1.13, − 0.46)	3.50e-06	rho	− 0.789941	< 2.2e-16
			lambda	0.878741	< 2.2e-16
***Interactive Model - Drugs***			S.D.	5.0317	
spreml(Cancer Rate ~ Age + Cigarettes * AUD * Cannabis + Analgesics + Cocaine			Log.Lik	− 2090.8060	
Cannabis	−1.81 (−3.22, − 0.39)	0.01231	phi	2.018035	0.0030
Age	−0.9 (−1.21, − 0.59)	1.81e-08	psi	0.61679	< 2.2e-16
			rho	−0.792062	< 2.2e-16
			lambda	0.88467	< 2.2e-16
***Interactive Model - Comprehensive***			S.D.	4.8427	
spreml(Cancer Rate ~ Age + Cigarettes * AUD * Cannabis + Analgesics + Cocaine + Income + Five Races)			Log.Lik	−2090.3930	
Age	−1 (−1.28, − 0.73)	4.04e-13	phi	1.751775	0.0003
Hispanic	−1.49 (− 2.56, − 0.42)	0.006247	psi	0.63281	< 2.2e-16
			rho	− 0.778829	< 2.2e-16
			lambda	0.88237	< 2.2e-16
***Interactive Cannabinoid Model - Comprehensive***					
spreml(Cancer Rate ~ Age + Cigarettes * THC * CBG * CBD + AUD + Income + Five Races)					
AUD	36.55 (9.15, 63.96)	0.0090	S.D.	5.1269	
CBG	2.18 (0.2, 4.16)	0.0312	Log.Lik	− 2079.543	
THC: CBG: CBD	−5.19 (−8.32, − 2.06)	0.0012	phi	2.152804	0.0015
THC: CBD	−17.59 (−28.21, −6.97)	0.0012	psi	0.579133	< 2.2e-16
THC: CBG	−21.24 (−33.23, −9.25)	0.0005	rho	−0.779342	< 2.2e-16
THC	−74.58 (−115.58, − 33.58)	0.0004	lambda	0.862257	< 2.2e-16
Age	−0.84 (−1.24, − 0.43)	4.61e-05			

Table 2 presents the results of various temporally and spatially lagged models. At 2 years lag cannabidiol is independently significant and the coefficient is positive. At six years lag cannabidiol is included in three terms with an overall net positive effect.

**Table 2:** Prostatic Cancer – Lagged Space – Time Models

Lagged Variables	Parameter			Model		
	Parameter	estimate (C.I.)	P	Coefficient	Value	***P***-Value
	***LAGGING WITH CANNABINOIDS***					
	***Comprehensive Interactive Model - 2 Temporal Lags***					
	spreml(Cancer Rate ~ Age + Cigarettes * THC * CBG * CBD + AUD + Analgesics + Cocaine + Income + Five Races)					
*Temporal Lags:*	Cocaine	80.28 (15.7, 144.86)	0.0148	S.D.	5.2086	
THC, 2	CBD	5.95 (0.76, 11.15)	0.0247	Log.Lik	− 1772.068	
Cannabidiol, 2	Analgesics	59.62 (6.42, 112.81)	0.0280	phi	2.689466	0.0004161
Cannabigerol, 2	Income	3.89 (0.36, 7.43)	0.0308	psi	0.583621	< 2.2e-16
	CBG	−5.07 (−9.71, − 0.44)	0.0318	rho	− 0.826646	< 2.2e-16
	Cigarettes: CBG	−62.38 (−108.32, −16.43)	0.0078	lambda	0.84905	< 2.2e-16
	Cigarettes	− 282.27 (− 484.13, −80.41)	0.0061			
	Cigarettes: CBD	−88.44 (−137.35, −39.53)	0.0004			
	Cigarettes: CBG: CBD	−20.64 (− 31.36, −9.92)	0.0002			
	Age	−0.72 (−1.09, − 0.35)	0.0001			
	***Comprehensive Interactive Model - 4 Temporal Lags***					
	spreml(Cancer Rate ~ Age + Cigarettes * THC * CBG * CBD + AUD + Analgesics + Cocaine + Income + Five Races)					
*Temporal Lags:*	Cocaine	0.0006	0.0006	S.D.	4.7498	
THC, 4	Hispanic	0.0012	0.0012	Log.Lik	− 1505.899	
Cannabidiol, 4	Age	2.18e-10	2.18e-10	phi	2.091523	0.0004814
Cannabigerol, 4				psi	0.652059	< 2.2e-16
				rho	−0.829187	< 2.2e-16
				lambda	0.876342	< 2.2e-16
	***Comprehensive Interactive Model - 6 Temporal Lags***					
	spreml(Cancer Rate ~ Age + Cigarettes * THC * CBG * CBD + AUD + Analgesics + Cocaine + Income + Five Races)					
*Temporal Lags:*	Cigarettes: THC: CBG	24.04 (14.72, 33.36)	4.29e-07	S.D.	5.1497	
THC, 6	Cigarettes: CBG	334.94 (176.94, 492.95)	3.26e-05	Log.Lik	− 1218.3530	
Cannabidiol, 6	Cigarettes	1080.16 (552.4, 1607.92)	6.03e-05	phi	2.710614	3.11e-05
Cannabigerol, 6	Cigarettes: CBG: CBD	49.06 (17.39, 80.73)	0.0024	psi	0.617539	< 2.2e-16
	Cigarettes: CBD	150.69 (45.25, 256.13)	0.0051	rho	−0.722522	< 2.2e-16
	Age	− 0.63 (−1.09, − 0.18)	0.0067	lambda	0.822562	< 2.2e-16
	CBG	−38.43 (−55.72, −21.13)	1.33e-05			
	THC	−53.27 (−77, −29.55)	1.07e-05			
	THC: CBD	−20.22 (− 28, − 12.44)	3.49e-07			
	***Comprehensive Interactive Model - 1 Spatial Lag***					
	spreml(Cancer Rate ~ Age + Cigarettes * THC * CBG * CBD + AUD + Analgesics + Cocaine + Income + Five Races)					
*Spatial Lags:*	Cigarettes: THC: CBD	−128.412 (− 202.52, − 54.31)	0.0006	S.D.	5.1497	
THC, 1	Cigarettes: THC: CBG: CBD	−38.166 (− 59.63, − 16.7)	0.0070	Log.Lik	− 2081.4700	
Cannabidiol, 1	Cigarettes: THC	−517.602 (− 800.36, − 234.85)	0.0053	phi	2.09673	3.96e-06
Cannabigerol, 1	Cigarettes: THC: CBG	−151.094 (− 232.66, −69.53)	0.0034	psi	0.593689	< 2.2e-16
	Age	− 0.949 (−1.34, − 0.56)	4.3e-05	rho	− 0.765839	< 2.2e-16
				lambda	0.867413	< 2.2e-16

Table 3 shows the final models from spatial and temporal lagging of various cannabinoids.

**Table 3:** Prostatic Cancer – Spatially and Temporally Lagged Space – Time Cannabinoid Models

Lagging	Parameter			Model		
Lagged Variables	Parameter	estimate (C.I.)	P	Coefficient	Value	***P***-Value
	***Spatiotemporal Lags***					
	***Comprehensive Interactive Model - 1 Spatial & 2 Temporal Lags***					
	***THC Temporally Lagged***					
	spreml(Cancer Rate ~ Age + Cigarettes * THC * CBG * CBD + AUD + Analgesics + Cocaine + Income + Five Races)					
*Spatial:*	Income	6.59 (3.22, 9.96)	0.0001	S.D.	4.6652	
THC, 1	Cocaine	1.82 (0.58, 3.06)	0.0039	Log.Lik	−1759.5510	
Cannabigerol, 1	Analgesics	2.54 (0.16, 4.92)	0.0365	phi	1.9006	0.0003446
Cannabidiol, 1	Hispanic	−1.74 (−3.13, − 0.35)	0.0139	psi	0.5811	< 2.2e-16
*Temporal:*	Cigarettes: THC: CBD	−128.33 (− 199.73, −56.93)	0.0004	rho	− 0.8096	< 2.2e-16
Cigarettes,2	Age	−0.82 (−1.27, − 0.37)	0.0004	lambda	0.8158	< 2.2e-16
AUD,2	Cigarettes: THC	−513.27 (− 786.94, − 239.59)	0.0002			
THC, 2	Cigarettes: THC: CBG: CBD	−39.99 (− 60.98, −19)	0.0002			
Analgesics, 2	Cigarettes: THC: CBG	−158.61 (− 238.74, − 78.49)	0.0001			
Cocaine, 2	THC	−2.9 (−3.93, − 1.87)	3.11e-08			
	***Comprehensive Interactive Model - 1 Spatial & 2 Temporal Lags***					
	***Cannabidiol Temporally Lagged***					
	spreml(Cancer Rate ~ Age + Cigarettes * THC * CBG * CBD + AUD + Analgesics + Cocaine + Income + Five Races)					
*Spatial:*	CBD	2.08 (1.19, 2.98)	5.20e-06	S.D.	4.7079	
THC, 1	Cocaine	2.56 (1.3, 3.81)	6.75e-05	Log.Lik	− 1758.7820	
Cannabigerol, 1	Income	5.5 (2.26, 8.75)	0.0009	phi	5.3487	1.83e-08
Cannabidiol, 1	CBD.Spatial	1.06 (0.03, 2.1)	0.0442	psi	0.6391	< 2.2e-16
*Temporal:*	AIAN	−32.1 (−60.98, −3.21)	0.0294	rho	−0.7110	5.65e-16
Cigarettes,2	THC: CBG: CBD	−0.43 (− 0.81, − 0.05)	0.0257	lambda	0.6854	< 2.2e-16
AUD,2	Hispanic	−2.33 (−3.69, −0.97)	0.0008			
Cannabidiol, 2	Cigarettes: THC: CBD	−163.72 (− 246.95, −80.48)	0.0001			
Analgesics, 2	Cigarettes: THC: CBG: CBD	−48.79 (−73.06, − 24.51)	8.17e-05			
Cocaine, 2	Cigarettes: THC	−651.36 (−967.53, − 335.2)	5.39e-05			
	Cigarettes: THC: CBG	−199.58 (− 291.7, − 107.47)	2.17e-05			
	Age	−1.18 (− 1.59, − 0.76)	2.36e-08			
*Spatial:*	***Comprehensive Interactive Model - 1 Spatial & 4 Temporal Lags***					
THC, 1	***THC Temporally Lagged***					
Cannabigerol, 1	spreml(Cancer Rate ~ Age + Cigarettes * THC * CBG * CBD + AUD + Analgesics + Cocaine + Income + Five Races)					
Cannabidiol, 1	THC	642.76 (165.3, 1120.23)	0.0083	S.D.	18.5723	
*Temporal:*	CBG: THC	181.1 (41.49, 320.71)	0.0110	Log.Lik	− 2045.3050	
Cigarettes,4	CBD: THC	146.56 (25.64, 267.49)	0.0175	phi	2.2917	0.0009418
AUD,4	CBG: CBD: THC	41.24 (5.57, 76.9)	0.0235	psi	0.6228	< 2.2e-16
THC, 4	Cocaine	1.42 (0.18, 2.65)	0.0247	rho	−0.8426	< 2.2e-16
Analgesics, 4	Cigarettes: CBG: CBD: THC	−192.44 (− 336.83, −48.04)	0.0090	lambda	0.8542	< 2.2e-16
Cocaine, 4	Cigarettes: CBD: THC	− 694.28 (−1189.26, − 199.3)	0.0060			
	Hispanic	−2 (−3.43, −0.58)	0.0060			
	Cigarettes: CBG: THC	− 837.5 (− 1403.64, − 271.36)	0.0037			
	Cigarettes: THC	− 3015.28 (− 4971.81, − 1058.74)	0.0025			
	Age	−1.06 (− 1.48, − 0.64)	7.82e-07			
*Spatial:*	***Comprehensive Interactive Model - 1 Spatial & 4 Temporal Lags***					
THC, 1	***Cannabidiol Temporally Lagged***					
Cannabigerol, 1	spreml(Cancer Rate ~ Age + Cigarettes * THC * CBG * CBD + AUD + Analgesics + Cocaine + Income + Five Races)					
Cannabidiol, 1	Cocaine	2.34 (1.21, 3.46)	4.53e-05	S.D.	4.7813	
*Temporal:*	THC: CBG	19.63 (6.78, 32.48)	0.0028	Log.Lik	− 1492.4380	
Cigarettes,4	THC	64.38 (22.13, 106.64)	0.0028	phi	2.2091	0.0002842
AUD,4	CBD	−1.05 (−2.04, −0.06)	0.0374	psi	0.5981	< 2.2e-16
Cannabidiol, 4	Cigarettes: THC: CBG: CBD: CBD.Spatial	−0.41 (− 0.78, − 0.05)	0.0272	rho	−0.8249	< 2.2e-16
Analgesics, 4	Cigarettes: THC: CBG	−83.75 (− 136.43, −31.07)	0.0018	lambda	0.8468	< 2.2e-16
Cocaine, 4	Hispanic	−2.26 (−3.63, − 0.9)	0.0012			
	Cigarettes: THC	−303.31 (−480.04, −126.57)	0.0008			
	Age	−1.06 (− 1.48, −0.64)	7.18e-07			

Various cannabinoids are shown to have both positive and negative effects on the prostate cancer rate. For each final model the net effect of cannabidiol is negative.

**Fig. 8:**
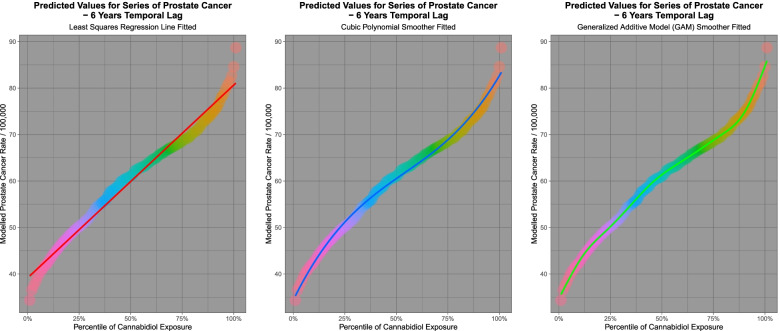
Modelled scaled output values from geospatial models of a comprehensive interactive prostate cancer model lagged to six years

It is of interest to consider the modelled behaviour of the predicted values as the percentile of cannabidiol exposure increases. For the purposes of examining model predictions the spatiotemporal model lagged to six years shown in Table 2 was chosen. Figure 8 shows the behaviour of the fitted outcomes from the model as a function of simultaneously increasing cannabidiol exposure. A line of best fit (panel A), a cubic regression line (in panel B) and a.

general additive model (in panel C) was fitted to these data. Table 4 presents the results of comparisons of the various percentiles from this model. One notes that the final column shows that the ratio of the various comparisons increases as a function of the increasing nature of the curve and its various inflections.

**Table 4:** Prostate Cancer - Percentile Rank Comparisons

Percentiles				Difference	Ratio
Low Percentile		High Percentile			
Rank	Value	Rank	Value		
10th Percentile	42.5469	90th Percentile	74.8319	32.2850	1.7588
5th Percentile	39.4475	95th Percentile	78.6431	39.1956	1.9936
1st Percentile	34.3401	99th Percentile	82.6379	48.2978	2.4065

Results of regression based upon the least squares regression lines, polynomials and GAM fitted curves is shown in Table 5. Anova tests demonstrated that the cubic model was significantly better than the linear model (Anova: F = 240.83, df = 2,97, *P* = 4.03 × 10^− 39^) and that the GAM model was also better than the linear model (Anova: F = 245.26, df = 3,96, *P* = 1.25 × 10^− 45^). These results show that the inflections in the curve are highly statistically significant and this is consistent with non-linearity of the effect, that is increasing effects at higher cannabidiol concentrations and an increasing rate of rise of the effect.

**Table 5:** Prostate Cancer – Predictive Regression Model Summaries

Linear Models								
**Parameter**					**Model**			
**Term**		**Estimate (C.I.)**		**P_Value**	**Adj.R.Squared**	**Standard Deviation**	**t-Value**	***P*** **-Value**
***Linear Model***								
Percentile		0.95 (0.92, 0.97)		2.52E-87	0.9811	3.8586	5185.354	2.52E-87
***Cubic Polynomial Model***								
First Order Percentile		277.86 (273.73, 281.98)		2.58E-111	0.9943	2.1028	5898.511	1.31E-109
Second Order Percentile		−10.41 (− 14.53, − 6.28)		3.15E-06				
Third Order Percentile		30.61 (26.49, 34.73)		3.91E-26				
GAM Model								
**Parameter**					**Model**			
**Term**	**Estimated Degress of Freedom**	**Residual Degrees of Freedom**	**Statistic**	***P*** **-Value**	**Log.Likelihood**		**Akaike Information Crierion**	**Bayesian Information Criterion**
Smoothened Percentile	8.8184	8.9902	8777.838	< 2.2E-320	− 137.9338		297.5044	325.7959

The applicable E-Values for these models are shown in Table 6. In particular one notes that the minimum E-Values for the cubic polynomial fit (5.59 × 10^51^ and 1.91 × 10^5^) are much higher than those for the linear model (1.79). As was noted above the polynomial is a much better fit to the modelled data.

**Table 6:** Prostate Cancer – E-Values of Predictive Regression Models

term	Estimate	Standard Error	Stanhdard Deviation	Relative Risk	E-Values
***Linear Model***					
Percentile	0.8193	0.0162	4.7448	1.25 (1.24, 1.26)	1.81, 1.79
***Cubic Polynomial Model***					
First Order Percentile	277.8563	2.1028	2.1028	1.66E+ 52 (2.79E+ 51, 9.84E+ 52)	3.31E+ 52, 5.59E+ 51
Third Order Percentile	30.6074	2.1028	2.1028	5.65E+ 05 (9.53E+ 04, 3.35E+ 06)	1.13E+ 06, 1.91E+ 05

### Ovarian cancer

**Fig. 9:**
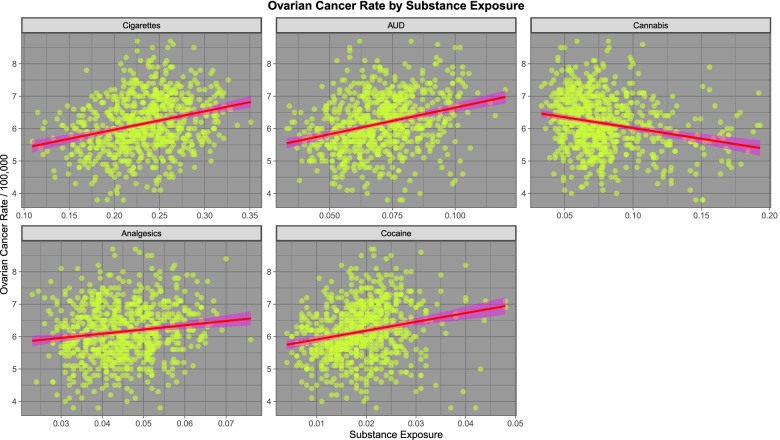
Relationship of ovarian cancer to various substance exposures

**Fig. 10:**
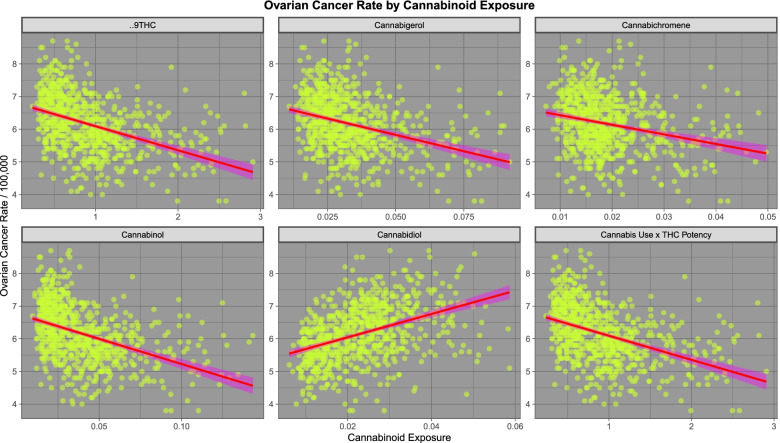
Relationship of various estimated cannabinoid exposures to ovarian cancer

We move next to consideration of ovarian carcinoma. As shown in Fig. 9 ovarian cancer shows a positive relationship with all five substances examined except cannabis. As seen in Fig. 10 ovarian carcinoma shows a positive relationship with cannabidiol but a negative relationship with other cannabinoids.

**Fig. 11:**
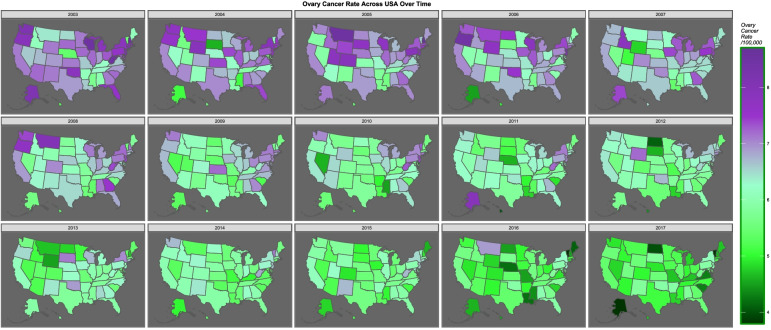
Map-graph of ovarian cancer rates across USA over time

**Fig. 12:**
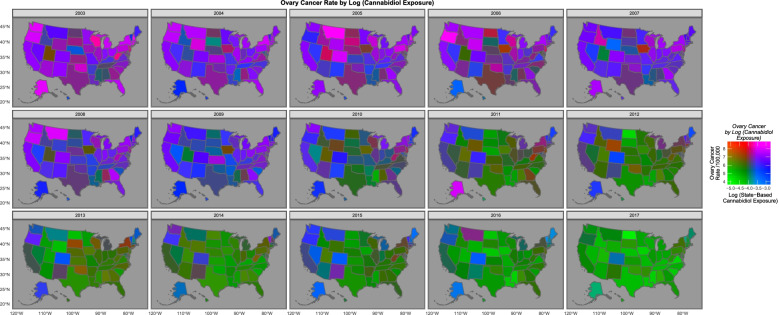
Bivariate map-graph of the relationship between cannabidiol use and the ovarian cancer across USA over time

Figure 11 shows the falling rate of ovarian cancer across USA over time. The bivariate relationship between cannabis use and ovarian cancer is shown map-graphically in Fig. 12.

Mixed effects models for ovarian cancer are shown in Supplementary Table 7 (Excel sheet “ST1 Ov lme”). Interestingly in additive models for drugs and for all covariates, cannabis is independently and positively predictive.

Supplementary Table 8 (Excel sheet “ST1 Ov lme Cannbd”)presents the results of comprehensive additive and interactive cannabinoid models. The three cannabinoids THC, cannabigerol and cannabidiol are noted to be significant in both models. Cannabidiol is independently significant with a positive coefficient in the interactive model.

The positive relationship between cannabidiol and ovarian cancer is confirmed by robust generalized linear regression in Supplementary Table 9 (Excel sheet “ST1 Ov SG”).

In the robust comprehensive interactive models in Supplementary Table 10 (Excel sheet “ST1 Ov SG Cannbd”) whilst the effects of cannabidiol are negative overall the effects of rising cannabinoid percentiles is positive.

At panel regression cannabis is both independently positive in its effects on ovarian cancer in additive models and has a positive effect overall in interactive models, as shown in Supplementary Table 11 (Excel sheet “ST1 Ov plm Intro”).

Supplementary Table 12 (Excel sheet “ST1 Ov plm Add”) shows a series of additive cannabinoid panel models lagged to 0, 2, 4, 6 and 8 years. One notes that at 2 and 8 years cannabidiol has a positive and independently highly significant effect (β-estimate = 1.84 (1.44, 2.23), *P* = 1.2 × 10^− 19^ and β-estimate = 8.51 (6.96, 10.07), *P* = 8.06 × 10^− 27^ respectively)

In interactive cannabinoid panel models cannabidiol is again positively related to ovarian cancer rates at both zero and two years lag (Supplementary Table 13, Excel sheet “ST1 Ov plm IR”)).

Table 7 shows the results of introductory temporospatial modelling. The effect of cannabidiol alone is again noted to be positive (β-estimate = 0.36 (0.3, 0.42), *P* <  2.2 × 10^− 16^).

**Table 7:** Ovarian Cancer – Introductory Space-Time Regression

Parameter			Model		
Parameter	estimate (C.I.)	P	Coefficient	Value	***P***-Value
***Cannabis Alone***			S.D.	0.6119	
***spreml(Cancer Rate ~ Cannabis)***			Log.Lik	− 719.3662	
Cannabis	−0.46 (− 0.57, − 0.35)	< 2.2e-16	phi	0.578764	5.73e-05
			rho	−0.786241	< 2.2e-16
			lambda	0.803723	< 2.2e-16
***THC Alone***			S.D.	0.6212	
***spreml(Cancer Rate ~ THC exposure)***			Log.Lik	−699.7751	
THC exposure	−0.36 (− 0.41, − 0.31)	< 2.2e-16	phi	0.534787	5.09e-05
			rho	−0.687793	< 2.2e-16
			lambda	0.7014	< 2.2e-16
***Cannabigerol Alone***			S.D.	0.6175	
***spreml(Cancer Rate ~ Cannabigerol exposure)***			Log.Lik	− 709.6908	
Cannabigerol exposure	−0.43 (−0.5, − 0.35)	< 2.2e-16	phi	0.557597	5.16e-05
			rho	−0.738106	< 2.2e-16
			lambda	0.754643	< 2.2e-16
***Cannabidiol Alone***			S.D.	0.6246	
***spreml(Cancer Rate ~ Cannabidiol exposure)***			Log.Lik	−709.5827	
Cannabidiol exposure	0.36 (0.3, 0.42)	< 2.2e-16	phi	0.58377	4.66e-05
			rho	−0.71922	< 2.2e-16
			lambda	0.746991	< 2.2e-16
***Additive Model - Drugs***					
***spreml(Cancer Rate ~ Age + Cigarettes + AUD + Cannabis + Analgesics + Cocaine***			S.D.	0.6667	
Analgesics	6.5 (0.56, 12.43)	0.0319	Log.Lik	− 682.9304	
AUD	4.82 (1.04, 8.59)	0.0123	phi	0.836607	3.34e-05
Cigarettes	3.97 (2.36, 5.57)	1.27e-06	rho	−0.690814	< 2.2e-16
Age	−0.06 (− 0.1, − 0.03)	0.0005	lambda	0.637209	< 2.2e-16
***Interactive Model - Drugs***					
spreml(Cancer Rate ~ Age + Cigarettes * AUD * Cannabis + Analgesics + Cocaine			S.D.	0.6630	
Cigarettes	2.73 (0.64, 4.83)	0.0104	Log.Lik	− 681.5923	
Analgesics	6.83 (0.89, 12.76)	0.0243	phi	0.810219	4.21e-05
Age	−0.05 (−0.09, − 0.02)	0.0055	rho	−0.683232	< 2.2e-16
Cigarettes: Cannabis: AUD	−6.85 (−11.26, −2.43)	0.0024	lambda	0.631604	< 2.2e-16
***Interactive Model - Comprehensive***					
spreml(Cancer Rate ~ Age + Cigarettes * AUD * Cannabis + Analgesics + Cocaine + Income + Five Races)					
Cigarettes	3.9 (2.04, 5.77)	4.19e-05	S.D.	0.6762	
Analgesics	6.42 (0.38, 12.45)	0.0373	Log.Lik	−674.869	
African	−0.14 (− 0.24, − 0.03)	0.0095	phi	3.3102	0.003983
Income	−0.62 (−1.07, − 0.16)	0.0083	rho	− 0.6737	< 2.2e-16
AIAN	−5.52 (−9.19, − 1.86)	0.0032	lambda	0.7279	< 2.2e-16
Age	−0.07 (− 0.11, − 0.03)	0.0005			
***Interactive Cannabinoid Model - Comprehensive***					
spreml(Cancer Rate ~ Age + Cigarettes * THC * CBG * CBD + AUD + Income + Five Races)					
Cigarettes	3.58 (1.58, 5.58)	0.0005	S.D.	0.6448	
CBG	0.69 (0.32, 1.07)	0.0003	Log.Lik	−672.8832	
Hispanic	0.15 (0.01, 0.29)	0.0303	phi	0.686632	4.17e-05
Cocaine	−8.04 (−15.8, −0.28)	0.0422	rho	−0.640042	1.43e-15
AIAN	−3.78 (−7.09, −0.46)	0.0256	lambda	0.597138	< 2.2e-16
Income	−0.73 (−1.21, − 0.26)	0.0023			
THC	−0.72 (−1, − 0.44)	3.87e-07			

As shown in Table 8 cannabis exposure is negatively associated with ovarian cancer.

**Table 8:** Ovarian Cancer – Lagged Space-Time Regression

Lagged Variables	Parameter			Model		
	Parameter	Estimate (C.I.)	P	Coefficient	Value	***P***-Value
	***LAGGING WITH CANNABIS***					
	***Temporal Lagging***					
	***Comprehensive Interactive Model - 2 Temporal Lags***					
	spreml(Cancer Rate ~ Age + Cigarettes * AUD * Cannabis + Analgesics + Cocaine + Income + Five Races)					
Cigarettes, 2	Caucasian	−0.78 (− 1.49, − 0.07)	0.0306	S.D.	0.6405	
AUD, 2	AIAN	−5.31 (− 8.92, − 1.71)	0.0039	Log.Lik	− 569.0211	
Cannabis, 2	African	−0.18 (− 0.29, − 0.08)	0.0006	phi	0.8173	8.97e-05
Analgesics, 2	Income	−0.92 (−1.32, − 0.53)	4.50e-06	rho	− 0.6447	7.27e-16
Cocaine, 2	Cannabis	−0.5 (− 0.69, − 0.31)	2.71e-07	lambda	0.6346	< 2.2e-16
	***Comprehensive Interactive Model - 4 Temporal Lags***			S.D.	0.5950	
	spreml(Cancer Rate ~ Age + Cigarettes * AUD * Cannabis + Analgesics + Cocaine + Income + Five Races)			Log.Lik	− 473.9722	
Cigarettes, 4	Cocaine	13.5 (5.36, 21.65)	0.0012	phi	0.5570	0.0002
AUD, 4	Cigarettes	2.34 (0.29, 4.4)	0.0257	rho	−0.5503	8.03e-08
Cannabis, 4	Cannabis	−0.37 (− 0.57, − 0.17)	0.0003	lambda	0.6351	< 2.2e-16
Analgesics, 4						
Cocaine, 4						
***Spatial Lagging***						
	***Comprehensive Interactive Model - 1 Spatial Lag***					
	spreml(Cancer Rate ~ Age + Cigarettes * AUD * Cannabis + Analgesics + Cocaine + Income + Five Races)					
Cannabis, 1	Cigarettes: Cannabis: AUD:	2.87 (1.57, 4.17)	1.44e-05	S.D.	0.6618	
	AIAN	−3.74 (−7.14, −0.33)	0.0313	Log.Lik	−674.5209	
	Cannabis	−0.45 (− 0.82, − 0.08)	0.0183	phi	0.8060	3.02e-05
	Age	−0.06 (− 0.11, − 0.02)	0.0042	rho	−0.6641	< 2.2e-16
	Cannabis	−0.16 (− 0.26, − 0.06)	0.0017	lambda	0.6062	< 2.2e-16
	Income	−0.7 (−1.11, − 0.29)	0.0008			
	***Spatiotemporal Lagging***					
*Spatial Lags:*	***Comprehensive Interactive Model - 1 Spatial & 2 Temporal Lags***					
Cannabis, 1	In this model Cannabis was considered as both a spatially and temporally lagged variable					
*Temporal Lags:*	spreml(Cancer Rate ~ Age + Cigarettes * AUD * Cannabis + Analgesics + Cocaine + Income + Five Races)					
Cigarettes, 2	Cigarettes	3.9 (2.15, 5.64)	1.21e-05	S.D.	18.7670	
AUD, 2	White	−0.97 (−1.68, − 0.25)	0.0082	Log.Lik	− 2419.9740	
Cannabis, 2	AIAN	−6.01 (−9.63, −2.39)	0.0011	phi	0.7901	0.0001
Analgesics, 2	African	−0.2 (− 0.31, − 0.1)	0.0002	rho	− 0.6287	2.11e-14
Cocaine, 2	Cannabis	−0.52 (− 0.71, − 0.32)	2.58e-07	lambda	0.6229	< 2.2e-16
*Spatial Lags:*	***Comprehensive Interactive Model - 1 Spatial & 4 Temporal Lags***					
Cannabis, 1	In this model Cannabis was considered as both a spatially and temporally lagged variable			S.D.	0.5877	
*Temporal Lags:*	spreml(Cancer Rate ~ Age + Cigarettes * AUD * Cannabis + Analgesics + Cocaine + Income + Five Races)			Log.Lik	−474.4161	
Cigarettes, 4	Cocaine	14.43 (6.66, 22.21)	0.0003	phi	0.5226	0.0001
AUD, 4	Income	−0.47 (−0.94, 0)	0.0492	rho	−0.5685	1.27e-08
Cannabis, 4	Cannabis	−0.32 (− 0.54, − 0.1)	0.0050	lambda	0.6437	< 2.2e-16
Analgesics, 4						
Cocaine, 4						

Table 9 presents the results of spatial models lagged to 2, 4 and 6 years. In the first two models cannabinoids have a negative effect on ovarian cancer incidence. When lagged to 6 years cannabinoids in general, and cannabidiol in particular, has an overwhelmingly positive effect on ovarian cancer incidence.

**Table 9:** Ovarian Cancer – Lagged Cannabinoid Space-Time Regression

Lagged Variables	Parameter			Model		
	Parameter	estimate (C.I.)	P	Coefficient	Value	***P***-Value
*Temporal Lags:*	***LAGGING WITH CANNABINOIDS***					
THC, 2	***Comprehensive Interactive Model - 2 Temporal Lags***					
Cannabidiol, 2	spreml(Cancer Rate ~ Age + Cigarettes * THC * CBG * CBD + AUD + Analgesics + Cocaine + Income + Five Races)					
Cannabigerol, 2	Income	− 0.57 (−1.03, − 0.11)	0.0157	S.D.	0.6168	
Cigarettes, 2	THC	−0.32 (− 0.42, − 0.23)	1.64e-10	Log.Lik	− 574.7914	
AUD, 2				phi	0.587232	5.71e-05
Analgesics, 2				rho	−0.581409	5.83e-10
Cocaine, 2				lambda	0.628577	< 2.2e-16
*Temporal Lags:*	***Comprehensive Interactive Model - 4 Temporal Lags***					
THC, 4	spreml(Cancer Rate ~ Age + Cigarettes * THC * CBG * CBD + AUD + Analgesics + Cocaine + Income + Five Races)					
Cannabidiol, 4	Cocaine	9.14 (0.28, 18)	0.0433	S.D.	0.3489	
Cannabigerol, 4	Cigarettes: THC: CBG	−1.16 (−1.91, −0.42)	0.0021	Log.Lik	− 460.3743	
Cigarettes, 4	Cigarettes: THC	−6.09 (−8.87, −3.31)	1.79e-05	phi	0.50581	9.48e-05
AUD, 4				rho	−0.47394	3.27e-05
Analgesics, 4				lambda	0.526539	7.87e-14
Cocaine, 4						
	***Comprehensive Interactive Model - 6 Temporal Lags***					
*Temporal Lags:*	spreml(Cancer Rate ~ Age + Cigarettes * THC * CBG * CBD + AUD + Analgesics + Cocaine + Income + Five Races)					
THC, 6	Cigarettes: THC: CBD	1.93 (1.07, 2.78)	9.96e-06	S.D.	0.5552	
Cannabidiol, 6	White	1.31 (0.53, 2.09)	0.0010	Log.Lik	−359.3580	
Cannabigerol, 6	Hispanic	0.24 (0.09, 0.39)	0.0016	phi	0.311493	0.0009978
Cigarettes, 6	Cocaine	16.36 (5.53, 27.19)	0.0031	rho	−0.25786	0.0821654
AUD, 6	THC: CBG: CBD	0.07 (0.02, 0.12)	0.0049	lambda	0.40592	8.75e-05
Analgesics, 6	AUD	−6.67 (−11.56, −1.79)	0.0074			
Cocaine, 6	Cigarettes: CBG: CBD	−0.24 (−0.4, − 0.09)	0.0025			

Spatiotemporally lagged models are presented in Table 10. The effect of cannabinoids in these models is negative.

**Table 10:** Ovarian Cancer – Spatially and Temporally Lagged Space-Time Regression

Lagged Variables	Parameter			Model		
	Parameter	Estimate (C.I.)	P	Coefficient	Value	***P***-Value
	***Comprehensive Interactive Model - 1 Spatial Lag***					
	spreml(Cancer Rate ~ Age + Cigarettes * THC * CBG * CBD + AUD + Analgesics + Cocaine + Income + Five Races)					
*Spatial Lags:*	White	47.91 (20.68, 75.13)	0.0006	S.D.	0.4125	
THC, 1	Age	−2 (−3.46, − 0.55)	0.0070	Log.Lik	−672.3628	
Cannabidiol, 1	Cannabigerol: Cannabidiol	− 0.94 (− 1.59, − 0.28)	0.0053	phi	4.2682	1.83e-08
Cannabigerol, 1	THC: Cannabigerol	−6.3 (− 10.51, − 2.08)	0.0034	rho	− 0.5883	2.59e-12
	THC	−31.25 (−46.22, − 16.28)	4.3e-05	lambda	0.6520	< 2.2e-16
*Spatial:*	***Comprehensive Interactive Model - 1 Spatial & 1 Temporal Lags***					
THC, 1	***THC Lagged both Temporally and Spatially***					
Cannabigerol, 1	spreml(Cancer Rate ~ Age + Cigarettes * THC * CBG * CBD + AUD + Analgesics + Cocaine + Income + Five Races)					
Cannabidiol, 1	Cigarettes	6.17 (3.38, 8.97)	1.54e-05	S.D.	0.6109	
*Temporal:*	Hispanic	0.16 (0.03, 0.29)	0.0162	Log.Lik	−621.5385	
Cigarettes,1	African	−0.11 (−0.21, − 0.02)	0.0201	phi	0.5509	8.38e-05
AUD,1	AIAN	−5.2 (−8.47, −1.94)	0.0018	rho	−0.6617	9.76e-16
THC, 1	Cigarettes: CBG: CBD	−0.28 (− 0.45, − 0.11)	0.0013	lambda	0.6109	< 2.2e-16
Analgesics, 1	Income	−0.83 (−1.3, − 0.36)	0.0006			
Cocaine, 1	THC	−0.22 (− 0.32, − 0.12)	1.07e-05			
*Spatial:*	***Comprehensive Interactive Model - 1 Spatial & 2 Temporal Lags***					
THC, 1	***THC Lagged both Temporally and Spatially***					
Cannabigerol, 1	spreml(Cancer Rate ~ Age + Cigarettes * THC * CBG * CBD + AUD + Analgesics + Cocaine + Income + Five Races)					
Cannabidiol, 1	CBG	0.77 (0.38, 1.16)	9.90e-05	S.D.	0.6190	
*Temporal:*	Income	−0.76 (−1.23, − 0.29)	0.0017	Log.Lik	−567.1623	
Cigarettes,2	THC	−0.52 (− 0.78, − 0.26)	7.78e-05	phi	0.5898	8.16e-05
AUD,2	THC	−0.33 (− 0.48, − 0.17)	2.67e-05	rho	−0.5512	2.44e-08
THC, 2				lambda	0.5790	< 2.2e-16
Analgesics, 2						
Cocaine, 2						
*Spatial:*	***Comprehensive Interactive Model - 1 Spatial & 4 Temporal Lags***					
THC, 1	***THC Lagged both Temporally and Spatially***					
Cannabigerol, 1	spreml(Cancer Rate ~ Age + Cigarettes * THC * CBG * CBD + AUD + Analgesics + Cocaine + Income + Five Races)					
Cannabidiol, 1	CBG	0.76 (0.22, 1.31)	0.0061	S.D.	0.5875	
*Temporal:*	Cocaine	0.25 (0.09, 0.42)	0.0023	Log.Lik	− 452.9861	
Cigarettes,4	THC: CBG	0.2 (0.05, 0.35)	0.0073	phi	0.4665	0.0001169
AUD,4	Cigarettes: THC: CBG: THC	−4.21 (−8.39, −0.03)	0.0482	rho	−0.4140	0.0006749
THC, 4	Cigarettes: THC: CBG: CBD: THC	−1.24 (−2.34, −0.15)	0.0261	lambda	0.4449	7.33e-08
Analgesics, 4	THC	−0.47 (− 0.62, − 0.33)	9.73e-11			
Cocaine, 4						

**Fig. 13:**
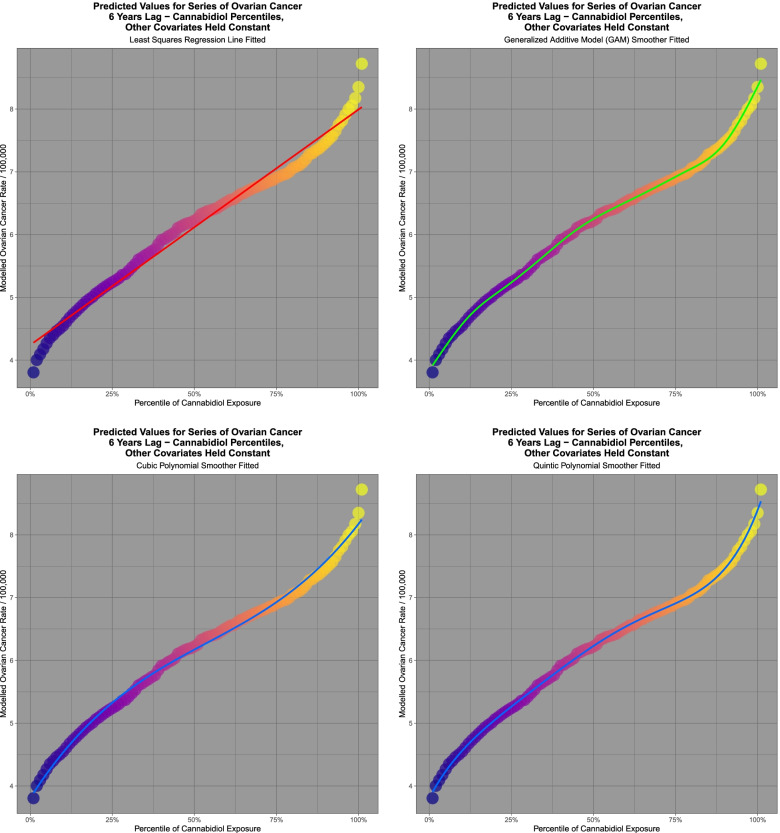
Modelled scaled output values from geospatial models of a comprehensive interactive ovarian cancer model lagged to six years

It is of interest to consider the effect of spatiotemporal modelling for ovarian carcinoma. Fig. 13 presents the results of predictive model output from the interactive spatial model at 6 lags shown in Table 9 of cannabinoids and ovarian cancer with 101 increasing percentiles of cannabidiol exposure. Again a sigmoidal curve shape is noted. Linear, cubic, quintic and GAM functions are fitted. Table 11 presents the results of the comparisons of the model values at varying cannabinoid percentiles and an increasing effect of rising cannabidiol concentrations is noted. The results of model regression are shown in Table 12.

**Table 11:** Ovarian Cancer – Predicted Model Percentile Values

Percentiles				Difference	Ratio
Low Percentile		High Percentile			
Rank	Value	Rank	Value		
10th Percentile	5.4089	90th Percentile	6.6647	1.2558	1.2322
5th Percentile	5.2884	95th Percentile	6.8129	1.5245	1.2883
1st Percentile	5.0897	99th Percentile	6.9683	1.8786	1.3691

**Table 12:** Ovarian Cancer – Predicted Regression Model Summaries

Linear Models								
**Parameter**					**Model**			
**Term**	**Estimate (C.I.)**		**P_Value**		**Adj.R.Squared**	**S.D.**	**t-Value**	***P*** **-Value**
***Linear Model***								
Percentile	0.016 (0.0157, 0.0166)		2.52E-87		0.9811	0.0656	5185.35	2.52E-87
***Cubic Polynomial Model***								
First Order Percentile	4.728 (4.657, 4.799)		2.58E-111		0.9944	0.0358	5898.511	1.31E-109
Second Order Percentile	−0.177 (−0.248, − 0.106)		3.15E-06					
Third Order Percentile	0.521 (0.449, 0.591)		3.91E-26					
***Quintic Polynomial Model***								
First Order Percentile	4.728 (4.698, 4.757)		1.19E-145		0.9991	0.0149	20,617.98	1.59E-142
Second Order Percentile	−0.177 (−0.206, − 0.147)		1.45E-20					
Third Order Percentile	0.520 (0.491, 0.550)		3.85E-56					
Fourth Order Percentile	0.244 (0.215, 0.274)		1.43E-29					
Fifth Order Percentile	0.208 (0.178, 0.237)		7.22E-25					
GAM Models								
**Parameter**					**Model**			
**Term**	**Estimated Degrees of Freedom**	**Residual Degrees of Freedom**	**statistic**	**P_Value**	**Log.Likelihood**	**Aliake Information Criterin**	**Bayesian Information Criterion**	
Smoothened Percentile	8.8097	8.9893	8777.844	1.46E-19	273.484	−525.3486	−497.0799	

Model comparison with anova tests confirm that the cubic fit is better than the linear fit (Anova: F = 118.17, df = 2,97, *P* = 2.89 × 10^− 27^), the quintic fit is better than the cubic fit (Anova: F = 233.77, df = 2,95, *P* = 3.44 × 10^− 38^), and the GAM model is better than both the linear fit (Anova: F = 177.85, df = 7.810, 914.19, *P* = 1.81 × 10^− 52^) and the cubic fit (Anova: F = 58.441, df = 5.81, 91.19, *P* = 5.91 × 10^− 29^). These results show that the inflections in the curves are statistically highly significant and explain the increasing acceleration of the effect of cannabidiol exposure on ovarian cancer incidence as the cannabidiol exposure rises, indicating a strong power function effect with rising dose.

Table 13 presents the E-Values applicable to the linear, cubic and quintic fitted functions for cannabidiol exposure, all of which are highly signifcant. Minimum E-Values range up to 1.92 × 10^− 125^ in this table for the quintic function.

**Table 13:** Ovarian Cancer – E-Values of Predicted Regression Models

Term	Estimate	Stamdard Error	Standard Devaition	Relative Risk	E-Values
***Linear Model***					
Percentile	0.0161	0.0002	0.0656	1.251 (1.243, 1.258)	1.81, 1.79
***Cubic Polynomial Model***					
First Order Percentile	4.7280	0.0357	0.0357	1.66E+ 52 (9.40E+ 50, 2.93E+ 53)	3.32E+ 52, 1.88E+ 51
Third Order Percentile	0.5208	0.0357	0.0357	5.65E+ 05 (3.21E+ 04, 9.98E+ 06)	1.13E+ 06, 6.41E+ 04
***Quintic Polynomial Model***					
First Order Percentile	4.7283	0.0149	0.0149	5.69E+ 125 (9.60E+ 124, 3.38E+ 126)	1.13E+ 126, 1.92E+ 125
Third Order Percentile	0.5208	0.0149	0.0149	7.12E+ 13 (1.21E+ 13, 4.23E+ 14)	1.43E+ 14, 2.40E+ 13
Fourth Order Percentile	0.2446	0.0149	0.0149	3.19E+ 06 (5.39E+ 05, 1.89E+ 07)	6.39E+ 06, 1.08E+ 06
Fifth Order Percentile	0.2084	0.0149	0.0149	3.48E+ 05 (5.87E+ 05, 2.06E+ 06)	6.96E+ 05, 1.17E+ 05

## Discussion

### Main results

As it was demonstrated in the first and second papers in this series [66, 67] that prostate and ovarian cancers were closely associated with cannabidiol exposure these tumours were explored in more analytical detail by way of the present exemplary analyses. The strong bivariate relationships observed were robust to adjustment in comprehensive interactive inverse probability weighted mixed effects, robust generalized and panel models and also in space-time analyses. In selected geospatial models for these two tumours polynomial minimum E-Values ranged up to 5.59 × 10^59^ and 1.92 × 10^125^. Moreover the dose-response relationships between rising modelled cannabidiol exposure and increasing cancer incidence was strongly non-linear with general additive model spline curves fitting the predicted data much better than linear models at significance levels of 1.25 × 10^− 45^ and 1.81 × 10^− 52^ respectively. This was strong evidence of a supra-linear sigmoidal power-function relationship with cancerogenesis.

We are very concerned at the supra-linear sigmoidal shape of the cannabinoid dose-oncogenesis response curve demonstrated in both tumours examined by predictive spatiotemporal modelling. Its direct corollary is that rising levels of cannabinoid exposure will be met by an inordinate increase in carcinogenesis. From the findings with AML and other pediatric cannabis-related tumours [11, 17–19, 96–99] real concerns exist that this may lead to a multigenerational epidemic of cancer. This view is closely concordant with a recent report describing cannabis exposure as a primary driver of USA pediatric cancers [100] and of the commonest cancer of childhood acute lymphoid leukaemia [28]. From the very clear findings with testicular cancer it would appear that the usual course of oncogenesis may be greatly accelerated [101].

The strong bivariate relationships reported herein and in the accompanying reports [66, 67] demonstrate that the cannabinoid-cancer relationships are robust to adjustment, fulfil quantitative epidemiological criteria for causality, and for prostate and ovarian cancer demonstrate a supra-linear sigmoidal dose-response relationship with carcinogenic outcomes so that rising doses of cannabinoid exposure generate disproportionate tumorigenic outcomes. Rather than prostate and ovarian cancer being outliers, our unpublished analyses to date show that the observations made on these cancers, particularly in relation to supra-linear sigmoidal dose-response exposure-oncogenic outcome relationships can also be found for many other tumours (manuscript in preparation). In this context the wide distribution and free availability of many cannabinoids including cannabidiol is of particular concern not only for the effect on the users, but as shown by ALL which is primarily a paediatric tumour [28], on subsequent generations who are exposed indirectly through parental access and presumably via gametotoxic, genotoxic and epigenotoxic pathways.

### Prostate cancer summary

Terms including THC, cannabigerol and cannabidiol are significant in final comprehensive interactive mixed effects models (Supplementary Tables 1 and 2). Cannabidiol is independently significant in comprehensive additive robust generalized linear model (Supplementary Table 3). In an interactive comprehensive robust generalized linear model the effects of cannabinoids THC, cannabigerol and cannabidiol were overwhelmingly positive (Supplementary Table 3).

In a series of lagged additive panel models cannabidiol was independently significant with positive coefficients at zero, four and six years (Supplementary Table 5). In a series of comprehensive interactive panel models cannabidiol was independently significant at zero and two years lag (Supplementary Tables 5 and 6).

Cannabidiol by itself was geospatiotemporally positively associated with prostate cancer rates (Table 1). In interactive geospatiotemporal models CBD was significantly positively associated with prostate cancer rates at 2 and 6 lags (Table 2). In an interactive spatiotemporal model with spatial and temporal lagging where cannabidiol was temporally and spatially lagged, cannabidiol was independently significantly associated with prostate cancer rates (Table 3). The cannabinoids THC, cannabigerol and cannabidiol are independently significantly associated with prostate cancer rates in comprehensive interactive space-time models (Tables 2 and 3).

Examining the space-time model lagged to 6 years one notes that the predictive values for increasing percentiles of cannabidiol exposure show a strong positive upward trend, and that the curve has obvious inflections making the cubic and GAM fits much better fits to the predicted model values. Inflections and supra-linear sigmoidality are highly statistically significant.

Hence in all pseudorandomized and geospatial models cannabinoids and cannabidiol are significantly associated with prostate cancer including positive coefficients in final comprehensive interactive models.

### Ovarian cancer summary

Cannabinoids are predictive in both additive and comprehensive mixed effects models (Supplementary Table 7). Cannabidiol is independently positively predictive in an interactive mixed effects model (Supplementary Table 8). In a robust generalized linear comprehensive interactive model cannabidiol is independently positively significant (Supplementary Table 10).

At 2 and 8 lags cannabidiol is independently and positively significant in lagged additive panel models (Supplementary Table 12). At zero and 2 years of lag terms including cannabidiol are positively significant in interactive panel models (Supplementary Table 13). In space-time models cannabidiol considered alone is positively significant (Table 7). Terms including cannabidiol are significant and positive at 6 lags (Table 8).

It is possible to consider ovarian cancer as a lagged function of increasing cannabidiol and cannabinoid concentrations. Inflections in the dose-response relationship curve strongly indicate that the relationship is supra-linear, sigmoidal and a non-linear power function of the percentile cannabidiol exposure.

Hence in all models cannabinoids and cannabidiol are significantly associated with ovarian cancer including positive coefficients in final comprehensive interactive models.

### Interpretation

#### Causal assignment

E-values have been used extensively in the present report. In the literature E-Values greater than 1.25 are said to be linked with causality [91]. It is worth noting that the minimum E-Value for the association between tobacco smoke and lung cancer is 9. This places the greatly elevated E-Values highlighted in this report in a proper context. The methodology employed here has also been validated *en passant* in that many tobacco-related cancers including lung, colorectum, all cancer, vulva and vagina, penis, bladder, oropharynx and esophagus, were correctly identified as such by the methodology adopted. Further age was correctly identified as a major risk factor for prostate cancer in the regression models.

Our regression modelling used inverse probability weighting in all mixed effects, robust generalized and panel regression models. This is the method of choice for application in observational studies to even out an exposure of interest across experimental groups and create a pseudo-randomized cohort from which causal inferences can properly be drawn.

#### Mechanisms

Central to any causal consideration of the relationship between cannabinoid exposure and carcinogenicity is the pivotal issue of the biological pathways by which cannabinoids might exert any oncogenic activities. This section is intended to be read alongside similar mechanistic discussions in the first and second papers in this series.

The subject of cannabinoids and cancer is too large to be reviewed in detail here. This and related subjects have been described in several other publications to which the interested reader is referred [45, 102–118]. Our intention here is merely to make some observations which are of particular interest and illustrate how all these seemingly disparate observations may present a coherent conceptual framework of cannabinoid-related carcinogenesis.

Rather than addressing prostatic and ovarian carcinogenesis specifically the present mechanistic discussion will focus on general oncogenic activities of cannabinoids in many tissues overall, and will touch on ovarian and germ cell oncogenesis where this is appropriate. This section will follow an outline. First a hierarchy of mechanistic considerations will be briefly reviewed proceeding from germ cells (eggs and sperm) to chromosomes and DNA.

### Germ cells

#### Sperm

The luminal concentration of lipophilic testosterone in the seminiferous tubules is known to be 100 times higher than that in the serum and it is maintained at these high levels in part by the blood testis barrier for which the morphological basis is the tight junctions between the supporting Sertoli cells which hold and cradle and nurture the developing spermatids [119]. Anandamide, one of the major endocannabinoids, is similarly concentrated in seminiferous tubules 12.0 + 2.1 nM [120] (compared to 5.7 + 0.9pM in serum [121]) where it acts to inhibit sperm activation, acrosomal reaction and swimming and metabolism by inhibiting mitochondrial respiration [65, 122, 123]. This makes sense because the sperm has limited metabolic reserves and penetration of the gransulosa cells and zona pellucida surrounding the oocyte is very difficult and requires hyperactivation of sperm motility in the context of the acrosomal reaction which releases digestive enzymes into the thick proteoglycan layers surrounding the egg. Cannabinoids are also suppressive to the hypothalamic release of LHRH, to LH release and to testicular Leydig cell endocrine function and thus acute serum testosterone levels [124, 125].

Indeed cannabinoids in testicular and male reproductive tissue have been noted to have many actions including affecting DNA fragmentation, sperm DNA packing, modification of sperm histones to sperm-specific variants which facilitate their replacement by protamines which are themselves tightly packed and heavily disulphide-linked cores for DNA wrapping, DNA nicking, DNA repair, protection of DNA, and thus nuclear size determination [122, 126].

Sperm have a series of specialized histones which make the genome more accessible and facilitate their replacement by protamines which allow much tighter DNA packing [127]. Interestingly in sperm 5–10% of histones remain in place and are not replaced by protamines which is one mechanism by which transgenerational epigenetic inheritance occurs [128]. In one study differential histone retention was only manifested in the F3 (grandchildren) generation [128].

#### Oocytes

Cannabinoids are found in the midcycle Graafian follicle fluid and the midcycle oviduct fluid [122, 126].

Polycystic ovarian syndrome (PCOS) is a clinical syndrome characterized by menstrual irregularity, excess androgens and sometimes ovarian cysts. It often accompanies obesity, may be complicated by systemic inflammation, impaired fertility and insulin resistance and may be complicated by endometrial carcinoma [129]. It is believed to have an heritable component. A fascinating recent paper showed that the ovary itself was involved in the dysregulated metabolic state and immune activation and that this was transmissible to a subsequent generation of mice via a hypomethylated DNA methylome [130]. DNA hypomethylation has also been demonstrated in the offspring of mice prenatally exposed to cannabis [117]. A characteristic gene signature was observed including Robo1, CDKN1, HDC1, IGFBPL1 and IRST4 in both mouse F1 offspring and daughters of human PCOS patients. Supplementation of the mice with a methyl donor S-adenosyl-methionine (SAM) rescued and reversed these changes [130]. Robo is also a key brain morphogen which directs the exuberant neocortical outgrowth in human infants [131] and the Robo-slit system has been shown to be inhibited by cannabinoids [132].

Certain features of this syndrome are reminiscent of the changes seen in human females consuming cannabis including the impaired fertility and altered reproductive hormones [124]. Moreover cannabinoids have been shown to interact with Robo [132]. Like other tissues the ovary will undergo increased methylation of CpG islands in and near gene transcriptional start sites with age. Epigenetic changes are known to be largely impacted by metabolic processes as described above. Moreover age-related decline in ovarian mitochondrial respiratory function also occurs [133]. It has been shown that age-related ovarian follicular failure in mice could be rescued by dietary supplementation of coenzyme Q10 [134]. The interaction between epigenomic, metabolic and immune processes is well documented [130, 135–137].

#### Chromosomes

Chromosomal damage is increasingly recognized as a major cause of tumourigenesis generally [138–141].

As mentioned evidence of single stranded and double stranded breaks in chromatin after cannabis exposure have been provided by several classical studies including dramatic photomicrographs of chromosomes with obvious breaks and gaps in them [142–145]. Pictures of ring and long chains of four chromosomes have also been described [146]. Indeed Stenchever found that the rate of chromosomal breaks was 3.4% compared to 1.2% in control cells [145]. Evidence of whole genome doubling has also been presented [143, 144] which is of particular relevance to testicular cancer where this is known to occur as a major precursor genetic lesion [147, 148]. Leuchtenberger published dramatic photomicrographs showing obviously lagging chromosomes in metaphase and anaphase spreads of dividing human lung cells [143]. These are well known to be the morphological precursors of micronucleus formation [149].

Micronuclei are known to be a major engine of tumourigenesis and of birth defect induction when they occur in germinative cells [138–141, 149–154]. For this reason in vitro and in vivo micronucleus assays have been foundational in genotoxicity testing and are written into the OECD genotoxicity testing Guidelines 474 and 487 [150].

Micronuclei are believed to arise either from aneugens which break off pieces of the chromatid ends, or by clastogens which interfere with the action of the mitotic spindle and sister chromatid separation at anaphase [150]. A further mechanism has been described involving nuclear elongation [150]. It has recently been suggested that nuclear mobilization, elongation and deformity may be central to the mechanism by which cannabidiol induces micronucleus formation [150].

Cannabinoids including THC, cannabidiol, cannabinol and cannabidivarin have been well demonstrated to test positively in the micronucleus assay for many decades [146, 155–159]. Synthetic cannabinoids including AM-2201, UR-144, 5F-AKB-48, AM-2201-1C, CP-478497-C8, RCS4, XLR-11, APINAC, BB-22, JWH-018, JWH-018-CL and STS-135 also test positive in micronucleus assays [160–163].

Nuclear blebs and chromosomal bridges are known to be associated with micronucleus development [156] and have been described after THC exposure in lymphocytes and oocytes [60, 164]. Nuclear blebs and bridges are also seen often in association with cannabinoid exposure [150, 156].

Cannabis has long been known to test positively in the micronucleus assay [158, 159]. Micronuclei are believed to develop around chromosomes which become derailed from the mitotic spindle or lag behind and do not join it and then become encapsulated in their own nuclear envelope, where lacking the normal large complement of enzymes usually involved in DNA functions they are shattered by normal cell replicative processes [107, 115, 146, 149, 165–167]. Cannabis does this by interfering with tubulin synthesis since the rails of the mitotic spindle are made of microtubules which are essentially greatly elongated tubulin monomers [168]. For this reason cannabis has been designated as an indirect clastogen [115, 146, 165–167].

Importantly it has been shown that, along with many other proteins, tubulin undergoes a variety of post-translational modifications including glycosylation, which appear to affect its function, perhaps by giving it a subcellular address within the cell to target [169]. This “tubulin code” is believed to function somewhat like the “histone code”. Interruption of this glycation process interferes with flagellar function and makes sperm swim in a circular pattern so that linear progress towards an oocyte is impossible and fertility is greatly compromised. This is believed to be a major factor in male infertility [169].

This implies that protein glycosylation is not only a biomarker of various parameters but also a functional readout of cell’s protein state. This finding supports the previous call for protein glycosylation to be included along with epigenomic markers in a potential biomarker for cannabinoid exposure [109]. As cannabinoids penetrate increasingly into American society the need for a quantitative biomarker to objectively define past cannabinoid exposure for both clinical and epidemiological reasons becomes correspondingly greater.

#### DNA

It is well established that cannabinoids reduce cell growth and reduce synthesis of the macromolecules of life such as DNA, RNA and proteins including histones [30, 115, 146, 159, 165–167, 170–175]. Cannabinoids have been shown to inhibit cell growth and division in all three layers of the embryo as well as haemopoietic and mesenchymal stem cells and their derivatives in osteoblastic, adipoblastic, peripheral nerves and cutaneous adult tissues [171].

Cannabinoids including THC and cannabidiol have been shown to oxidize the purine and pyrimidine bases of DNA in a manner which is greatly amplified by metabolic activation which manifests due to the action of the cytochrome oxidizing system of the liver such as occurs normally in vivo [156, 176].

As mentioned evidence of single-stranded and double-stranded breaks in chromatin have been provided by several classical studies including dramatic photomicrographs of chromosomes with obvious breaks and gaps in them ((Leuchtenberger1971, Leuchtenberger1973, Gilmour1971, Stenchever1974)). Indeed Stenchever found that the rate of chromosomal breaks was 3.4% compared to 1.2% in control cells ((Stenchever1974)).

#### Retrotransposon activation

Gestational toxin exposure to arsenic is known to induce DNA hypomethylation in active retrotransposons mobilizing these mobile elements in the genome and leading to genomic instability, cancer birth defects and mental retardation which is transmissible to sperm and the following F1 generation [177].

DNA hypomethylation is also well described following cannabis exposure [110, 117] and has also been shown to be transmissible to sperm [110] and to the following generation where it may be detected in the Nucleus Accumbens of the brain [117].

It would appear feasible therefore that cannabinoid-related hypomethylation could similarly mobilize repeat elements in the human genome causing them to be replicated and to be inserted randomly into the genome destabilising its integrity in a manner which is known to lead to oncogenic destabilization.

Moreover some of the DNA material will leak into the cytoplasm where it will trigger innate immunity via the sensitive and powerful cyclic guanosyl monophosphate - cyclic adenosine monophosphate synthase (cGAS) – STimulator of INterferon Gamma (STING) pathway which is powerfully proinflammatory [178]. Inflammatory and oxidizing milieus directly stimulate retrotransposon activation which makes the “jumping genes jump” worse. Hence this sets up a positive feedback loop. This pathway has been shown to be a powerful driver of both innate immunity, tumour progression and aggressive metastatic behaviour [178–184].

cGAS-STING pathway is also strongly stimulated by micronuclei and their cytoplasmic rupture [181].

Such mechanisms may in part account for the numerous reports of aggressive cancers developing in young patients who consume large amounts of cannabis [185–188] and the many reports of widespread premalignant field changes in the tissues of the upper aerodigestive tracts [16, 20].

#### Generalization

We feel that our results are widely generalizable for a number of reasons. The datasets comprising the foundation of this analysis are a national census cancer data series, with age-standardization of cancer incidence rates performed by CDC [68], and a large nationally representative annual widely quoted survey of drug use data [189]. As noted above many of the present results have been reported elsewhere in sources external to this study. The present bivariate analysis is at once conceptually simple yet very powerful especially when paired with E-Value calculations. For prostate and ovarian cancer bivariate results were verified by further causal regression and space-time modelling which confirmed the bivariate results and demonstrated overall robustness to multivariable adjustment. One of the major result outputs from the present study was several E-Values which constitute one of the major pillars of causal inference. We feel that the large US datasets represent an ideal context within which to address the present concerns. In that the present results demonstrate causal relationships we are confident that they could be widely reproduced with the sole caveat that in nations where cannabis use is more widespread we would expect the findings to be stronger provided that the underlying datasets are sufficiently accurate.

## Strengths and limitations

This study has several strengths. A large national cancer census dataset was used. Age adjusted rates derived from CDC, SEER and NCI were employed. The drug dataset was taken from a large well-validated nationally representative dataset. The bivariate statistics were straightforward and combined with the power of E-values they were powerful to directly address. These studies were internally consistent and also and externally concordant with known data both on tobacco-related cancer and on cannabis-related cancer. For the inferential modelling three forms of inverse probability weighted regression were employed with broadly consistent results. Geospace-time regression was also used to capture the inherently spatiotemporal setting of the data including its inherently complex spatially and temporally autocorrelated error structure. Panelled graphs were used to allow the simultaneous display of results for direct comparison across many cancer types.

In common with most epidemiological studies individual level participant data was not available to it. State-level cannabinoid exposure had to be estimated as described as state level data itself was also not directly available. Another issue of considerable interest is the possible role of synthetic cannabinoids as genotoxins. In the absence of spatiotemporal data on this issue we are unable to comment on this increasingly important matter. However several lines of evidence suggest that they are likely to be implicated. Several recent studies implicate many cannabinoids in genotoxic activities [27, 28, 45, 100, 101, 156, 157, 190–192]. Long ago the genotoxic action was found to reside in the polycyclic olevitol nucleus of the cannabinoids with little modulation by the various side chains [29, 190]. And several other studies implicate synthetic cannabinoids in genotoxicity [160–163, 193–195]. Overall therefore we feel that this is a fertile and important area for further laboratory based investigation and epidemiological surveillance.

Furthermore this was also an ecological study. It is therefore potentially susceptible to the short-comings typical of ecological studies including the ecological fallacy and selection and information biases. Within the present paper we began to address these issues with the use of E-values in all Tables. This issue is further addressed by the detailed pathophysiological mechanisms which have been described above, by mention of other countries where many of the same findings have been made, and with the use of inverse probability weighting in multiple regression models and further extensive application of E-values in Parts 2 and 3 of the present series of papers.

## Conclusion

Strong bivariate relationships between cannabidiol exposure and prostate and ovarian cancer previously reported [66, 67] were confirmed to be robust to multivariable adjustment by mixed, panel, robust and spatiotemporal regression modelling. Mathematical modelling of the relationship between increasing percentiles of cannabidiol exposure and prostate and ovarian cancer demonstrated strong evidence of a supra-linear sigmoidal relationship between rising cannabidiol exposure and cancer incidence such that increases in community cannabidiol exposure can be predicted to greatly and disproportionately increase tumour incidence. The implication of both prostate and ovary (and also testicular in [3, 8, 10, 66, 101, 148, 196, 197]) cancers in this oncogenic portrait carries very grave implications for community transmission of mutagenic and oncogenic genotoxicity from both parental germ lines to subsequent generations. Further work to investigate these themes in more detail and increased depth and by groups working in related laboratory fields and epidemiological and statistical methodology is strongly indicated. The present study clearly highlights the dangers of allowing increased cannabinoid penetration into the community not only in terms of its relationship to adult carcinogenesis but also in terms of heritable and paediatric cancerogenesis and transgenerational transmission of mutagenic and oncogenic genotoxicity and epigenotoxicity and clearly demonstrates supra-linear quasi-exponential dose- oncogenic-response kinetics in population health profiles. Such results strongly underscore the likely risks of increased cannabinoid penetration into the food chain which at the time of writing has not been formally studied. The clear implication from the present work and its accompanying reports [66, 67] is that community penetration of cannabinoids should be carefully restricted not only as a matter of public health and safety including importantly integrity of the food chain, but also as a non-negotiable investment in the genomic health and onco-protection of multiple coming generations in a manner precisely analogous to that of all other seriously genotoxic agents. Particular concerns relate to the movement of increasing sections of the community into higher dose ranges of cumulative cannabinoid exposure in the context of exponentiation of genotoxic dose-responses in higher dose ranges which has now been convincingly demonstrated both in the laboratory and in epidemiological studies of human populations.

## Supplementary Information

**Additional file 1.**

## Data Availability

All data generated or analysed during this study are included in this published article and its supplementary information files. Data has been made publicly available on the Mendeley Database Repository and can be accessed from this URL 10.17632/dt4jbz7vk4.1.

## Abbreviations

AEA: Anandamide
AIAN: American Indian / Alaska Native
ALL: Acute Lymphoid Leukaemia
AML: Acute Myeloid Leukaemia
AUD: Alcohol Use Disorder
CB1R: Cannabinoid Type 1 Receptor
CBC: Cannabichromene
CBD: Cannabidiol
CBG: Cannabigerol
CBN: Cannabinol
CDC: Centers for Disease Control, Atlanta, Georgia
cGAS-STING: Cytoplasmic GMP-AMP Synthase and the Stimulators of Interferon Gamma
CLL: Chronic Lymphoid Leukaemia
CML: Chronic Myeloid Leukaemia
DEA: Drug Enforcement Agency
E-Value: Expected Value
FOXM1: Forkhead Box M1
GC: Germinal Centre
IDO2: Indoleamine 2,3 Dioxygenase
mEV: Minimum E-Value (2.5% Threshold Level)
NCI: National Cancer Institute
NHL: Non-Hodgkins Lymphoma
NHPI: Native Hawaiian / Pacific Islander
NPCR: National Program of Cancer Registries
NSDUH: National Survey of Drug Use and Health
SAMHDA: Substance Abuse and Mental Health Services Administration
SAMHSA: Surveillance Epidemiology and End Results Program
SEER: Surveillance epidemiology and End Results Program
THC: Δ9-tetrahydrocannabinol
